# The clinical relevance of the metabolism of prostate cancer; zinc and tumor suppression: connecting the dots

**DOI:** 10.1186/1476-4598-5-17

**Published:** 2006-05-15

**Authors:** Leslie C Costello, Renty B Franklin

**Affiliations:** 1 Department of Biomedical Sciences, Dental School, University of Maryland, Baltimore, Maryland, USA

## Abstract

**Background:**

The genetic and molecular mechanisms responsible for and associated specifically with the development and progression of malignant prostate cells are largely unidentified. In addition, despite its implication in virtually all malignant cells, the role of altered cellular metabolism as an essential factor in prostate malignancy has been largely ignored. Moreover, the intermediary metabolism of normal prostate as well as malignant prostate cells is among the least studied and most poorly understood of all mammalian cells. Some important factors, especially the role of zinc, have been identified and implicated in the development and progression of prostrate malignancy. In this review, we provide a current and updated integrated assessment of the relationships of intermediary metabolism in normal prostate and in prostate cancer. The experimental and clinical evidence that leads to the formulation of concepts of normal and malignant prostate metabolism is presented. The evidence for a concept of zinc as a tumor suppressor agent and *Zip1 *zinc transporter as a tumor-suppressor gene is described.

**Results:**

The specialized function of the normal prostate glandular epithelium to produce and secrete enormously high levels of citrate involves and requires unique intermediary metabolism activities that are not generally associated with other normal mammalian cells. The accumulation of zinc by these cells is an essential factor in this unique metabolic relationship. In malignancy, the normal zinc-accumulating citrate-producing epithelial cells are metabolically transformed to citrate-oxidizing cells that lose the ability to accumulate zinc. A genetic alteration in the expression of ZIP1 zinc transporter is associated with this metabolic transformation. These genetic/metabolic relationships have important consequences on citrate-related metabolism, bioenergetics, cell proliferation and invasive capabilities of the malignant cells, which result in tumor-suppression characteristics.

**Conclusion:**

The genetic/metabolic relationships in normal prostate glandular epithelium are driven by the unique function to accumulate and secrete citrate. The genetic/metabolic transformation of the prostate malignant cells is driven by the metabolic/bioenergetic, growth/proliferative, and invasive/migration requirements of the malignant process. Zinc is critical to these relationships. An understanding of these genetic/metabolic relationships provides new directions and opportunities for development of regimens for the prevention and treatment of prostate cancer. Important insight into the genetic/metabolic requirements of the prostate malignant process is now evolving. Most importantly at this time, an appreciation and recognition of the genetic/metabolic significance and implications in the development of prostate malignancy is imperative; and much needed research in this area is essential. Hopefully, this review will help to achieve these goals.

## Introduction

About 230,000 males are expected to be identified with prostate cancer in the next year that will result in about 30,000 deaths. Despite the extensive clinical and experimental studies over the recent decades, the pathogenesis of prostate cancer remains unknown. The genetic and molecular mechanisms responsible for and associated specifically with the development and progression of malignant prostate cells are largely unidentified [[1,2] for reviews]. Coupled with this is the largely ignored role of altered cellular metabolism as an essential factor in prostate malignancy; although transformations of metabolism are implicated in virtually all malignant cells [3-8]. The combination of genetic/molecular/metabolic relationships is required to identify critical events in the prostate malignancy process. Such studies are beginning to reveal an essential understanding of the development and progression of prostate cancer.

This review will provide a current and updated integrated assessment of the relationships of intermediary metabolism in normal prostate and in prostate cancer. Much of the description of important contributions of earlier investigators and early studies that should be recognized is presented in our previous reviews [8-12]; and will not be repeated except where relevant to the present discussion. We will describe the experimental and clinical evidence that leads to the formulation of concepts of normal and malignant prostate metabolism. Important gaps, unresolved issues, and areas of required future studies relating to prostate intermediary metabolism will be identified. The review will culminate in defining important genetic/metabolic events in the development and progression of prostate malignancy. The evidence for a concept of zinc as a tumor suppressor agent and *ZIP1 *zinc transporter as a tumor-suppressor gene will be described. The clinical/translational application of these relationships is also presented.

The totality of this presentation is intended first to be informative. Hopefully, the presentation will be stimulating and even provocative; so that more attention is directed to this subject matter and other investigators might become involved. Finally, we hope to convince the clinical and scientific community-at-large of the importance of metabolic relationships in the development and progression of prostate cancer, and the relevance to new approaches to detection, treatment, and perhaps prevention of prostate cancer.

### Axioms of relationships of cellular activity, cellular metabolism, and malignancy

The following are important generalizations that we consider to be axiomatic.

1). In all cells, the existing cellular intermediary metabolism provides the bioenergetic/synthetic/catabolic requirements that are essential for the manifestation of the cells' current activities (function, growth, proliferation).

2). When a cell's activity changes, its metabolism must also be adjusted consistent with any newly established bioenergetic/synthetic/catabolic requirements

3). Malignant cells exhibit a parasitic existence. They have no specialized function other than the activities essential for their generational propagation (growth and proliferation), which occurs at the expense of their host.

4). Malignant cells are derived from normal cells that have undergone a genetic transformation to a neoplastic cell phenotype that is endowed with malignant potential.

5). Manifestation of the malignant potential of the neoplastic cell necessitates alterations in its metabolism (ie a metabolic transformation) to provide the bioenergetic/synthetic requirements of malignancy.

6). In the absence of the metabolic transformation, the neoplastic cell will not progress to complete malignancy. Conversely, the metabolic transformation, in the absence of the genetic transformation to a neoplastic malignant cell, will not cause malignancy.

7). Common to all malignant cells is the metabolic requirement for de novo lipogenesis/cholesterogenesis for membraneogenesis that is essential for their proliferative existence.

**Figure 1:**
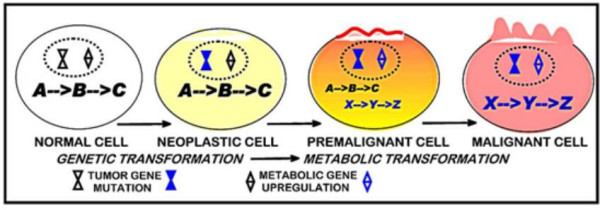
The concept of genetic/metabolic development of malignant cells.

These axioms define a relationship represented in figure 1 that we propose is applicable to all malignancies.

### Normal prostate function and associated intermediary metabolism: net citrate production

The human prostate gland is a complex organ comprised of different zones that have different embryological origin and different functional activities. The focus of this presentation is the peripheral zone, which comprises about 70% of the gland, and is the major functional component. Most importantly, this is the major region of malignancy. The primary function of the prostate gland is the production and secretion of prostatic fluid [8-12]. The major component of the prostatic fluid is its extraordinarily high concentration of citrate, which ranges from ~40–150 mM as contrasted with ~0.2 mM citrate in blood plasma (Table 1). Correspondingly, normal peripheral zone tissue citrate levels range from ~10,000–15,000 nmols/gram as contrasted with other tissues that generally contain ~250–450 nmols/gram.

**Table 1:** REPRESENTATIVE PROSTATE LEVELS

**(NMOLS/GRAM WWT)**	**CITRATE**	**ZINC**
NORMAL PERIPHERAL ZONE	12,000–14,000	3,000–4,500
MALIGNANT TISSUE	200–2000	400–800
OTHER TISSUES	250–450	200–400
PROSTATIC FLUID	40,000–150,000	8,000–10,000
BLOOD PLASMA	100–200	15

**Figure 2:**
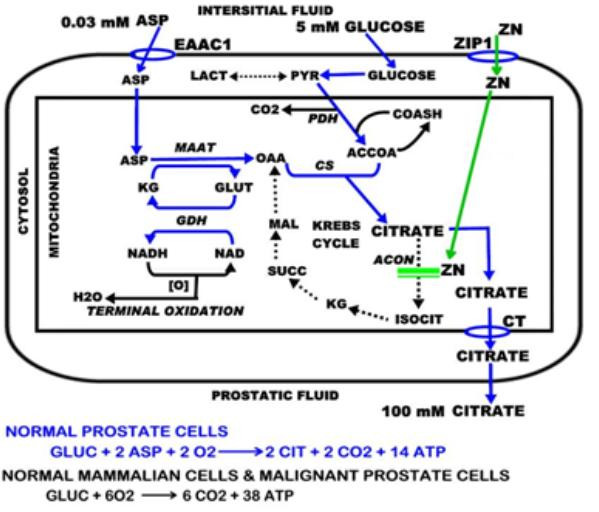
The metabolic pathway and bioenergetics of net citrate production in prostate cells.

The function of prostate citrate production is achieved by the activity of highly specialized glandular epithelial cells that have evolved for the capability to accumulate and secrete citrate, which we refer to as "net citrate production". This capability does not exist in other mammalian cells. Consequently the intermediary metabolism of these "citrate-producing" cells is "driven and modified" by their specialized function of net citrate production (figure 2). In typical mammalian cell intermediary metabolism, glucose utilization via glycolysis produces pyruvate which enters the mitochondria and is oxidized to acetyl coA. The acetyl coA condenses with OAA to produce citrate which is oxidized via the Krebs cycle with the regeneration of OAA. Consequently, in the oxidation of six-carbon citrate, two carbons are lost (CO2 production) and four carbons are conserved as OAA. In the citrate-producing prostate cells, all six carbons are removed from the metabolic pool; and citrate is an end-product of metabolism, rather than an utilizable intermediate. Therefore, citrate synthesis in these cells requires the availability of a source of acetyl coA and a source of OAA. The former is derived from glucose as described above. The latter is derived from aspartate. One must recognize the magnitude of prostate citrate production. The production of 1 ml of prostatic fluid, on average, involves the production of 100 umols citrate. To achieve this, 50 umols glucose and 100 umols aspartate will be utilized. The concentration of glucose and aspartate in blood plasma is 5 umols/ml and 0.03 umol/ml, respectively. Thus, a tremendous demand on the availability of glucose and aspartate is required for prostate citrate production.

To meet the precursor requirements for continued citrate synthesis, the prostate epithelial cells possess unique glucose and aspartate relationships. These cells, unlike most other mammalian cells, utilize glucose via a high aerobic glycolysis [13-17] that provides a high production of lactate←→pyruvate that is available for the production of acetyl coA.

For the production of OAA, aspartate is accumulated in the prostate cells and is transaminated via mAAT and coupled to GDH activity in a pathway that we define as the aspartate-glutamate-citrate pathway (figure 2). In these cells aspartate is an essential amino acid that is utilized; which is unlike most other mammalian cells that synthesize aspartate as a non-essential amino acid. Because the plasma aspartate concentration is low (0.03 mM) relative to its requirement for high citrate production, the prostate cells maintain a high cellular aspartate concentration (1.2 mM) availability for citrate synthesis [18]. This requires a capability of the prostate cells to extract and accumulate aspartate from circulation against the 40:1 concentration gradient. To achieve this, the prostate cells exhibit a high-affinity L-aspartate transport process [18-20]. We recently identified EAAC1 as the high-affinity aspartate transporter [21]; which is unique since EAAC1 in other mammalian cells generally operates as a high-affinity glutamate transporter rather than an aspartate transporter.

The relationships described above involve the metabolic adaptations that are essential to facilitate citrate synthesis. The accumulation of citrate is dependent upon its rate of synthesis versus its rate of utilization. The prevention of citrate oxidation by the prostate cells is the key relationship that is responsible for net citrate production. The prostate peripheral zone accumulates extremely high levels of zinc in the range of ~3000–5000 nmols/gram as compared to other tissues being ~200–400 nmols/gram. The cellular accumulation of zinc results in high levels of mitochondrial zinc that inhibit m-aconitase activity and citrate oxidation [22]. That zinc inhibition is the essential cause of limiting m-aconitase activity is evident from the recent observation that the level of m-aconitase enzyme is not low or changed in normal or malignant human and animal prostate cells [23] The inhibition of m-aconitase truncates the Krebs cycle at the first step of citrate oxidation; which provides the most efficient metabolic alteration for synthesized citrate to accumulate for secretion (figure 2).

Net citrate production has significant bioenergetic implications (figure 2). Compared to citrate-oxidizing cells that produce 38 ATP/complete oxidation of glucose; the citrate-producing prostate epithelial cells generate 14ATP/glucose utilized (or 20ATP if the NADH from the GDH reaction is oxidized by terminal oxidation). To accumulate citrate for secretion, these specialized cells sacrifice ~60% of the potential energy that could be derived from complete oxidation of glucose. In contrast to these highly specialized prostate cells, most mammalian cells cannot survive if m-aconitase activity and citrate oxidation are inhibited as is evident from the toxic effects of the m-aconitase inhibitor, fluorocitrate.

### Altered citrate and zinc relationships in prostate cancer: the metabolic transformation

It is now well established that citrate and zinc levels are markedly decreased in malignant versus normal prostate tissue. These biochemical changes occur early in the development of malignancy. Moreover, malignant prostate tissue virtually never retains the characteristic high levels of citrate and zinc found in normal peripheral zone. In the absence of high cellular zinc levels, m-aconitase activity is no longer inhibited and citrate oxidation proceeds via the Krebs cycle (figure 2). Thus we characterize the metabolic transformation as the transformation of zinc-accumulating citrate-oxidizing normal prostate epithelial cells to citrate-oxidizing cells that have lost the ability to accumulate zinc.

**Figure 3:**
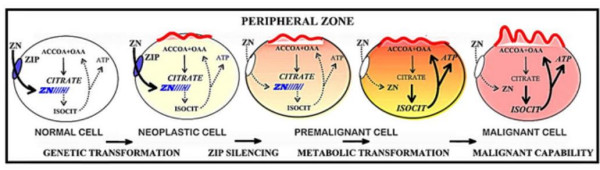
The concept of the role of zinc and altered citrate matabolism in the development of prostate malignancy.

An important benefit of this metabolic transformation is the bioenergetic relationship in that citrate oxidation provides the additional 24ATP from complete glucose oxidation that were lost by incomplete glucose oxidation in net citrate production. Thus, the malignant cells are bioenergetically more efficient than the specialized normal prostate epithelial cells. This is in contrast to the general tumor metabolism relationship in which the tumor cells are metabolically transformed to high aerobic glycolysis [3-7]. Figure 3 shows our concept of the genetic/metabolic transformation of the citrate-producing normal epithelial cell to the citrate-oxidizing malignant cell in the development of prostate malignancy.

### The metabolic role of citrate: oxidation and/or cytosolic acetyl CoA production

**Figure 4:**
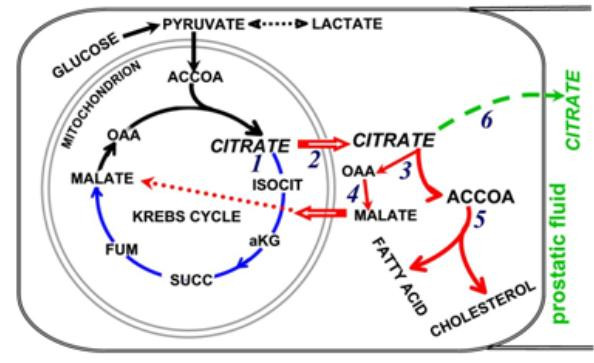
The pathways of citrate in mammalian cells. Pathway of citrate synthesis shown with black arrows is common to all cells. Pathway of citrate oxidation via the Krebs cycle is shown with blue arrows. Pathway of citrate export and utilization for lipogenesis/cholesterogenesis is shown with red arrows. Pathway of citrate secretion that occurs only in normal prostate cells is shown with green arrow.

In mammalian cells, citrate is synthesized in the mitochondria where it is either oxidized via the Krebs cycle or it is exported to the cytosol (figure 4). In the cytosol, the citrate is converted by ATP citrate lyase to acetyl coA, which is then utilized in lipogenesis/cholesterogenesis. In normal prostate cells, as already described, very little citrate is oxidized; so that essentially all the synthesized citrate is destined for export to cytosol. The cytosolic citrate is then secreted into prostate fluid. This leads to the expectation that ATP citrate lyase activity must be absent or very low in these prostate cells due to the absence of the enzyme or the existence of an inhibitor of the enzyme. Currently, reports concerning this relationship in normal prostate cells do not exist. Halliday et al [24] did infer that ATP citrate lyase activity was low in benign prostate glands and increased in malignant prostate glands; but direct studies of the enzyme were not conducted.

This requires another metabolic adaptation for the specialized function of citrate secretion. As shown in figure 4, the export of citrate is coupled to the import of a counter-ion, which is usually malate. The counter-ion is generated from the ATP citrate lyase production of OAA, which is absent in the normal citrate-producing prostate cell. Therefore, these cells must have an alternative source (currently unidentified) of a counter-ion for the export of citrate into the cytosol.

As already described, the malignant prostate cells undergo a metabolic transformation to citrate oxidation. However, for the malignant cells to proliferate they must also adapt their metabolism for the utilization of citrate for de novo lipid biosynthesis. The initiating steps in this pathway are the export of citrate into cytosol followed by its conversion to acetyl coA. Therefore, an essential metabolic adaptation in the malignant prostate cells must be an increase in ATP citrate lyase activity. Halliday et al [24] observed that a decrease in citrate levels correlates with a corresponding increase fatty acid levels in prostate malignant glands as contrasted with no such changes in neighboring BPH glands. They concluded that this is indicative of an increase in ATP citrate lyase activity in malignancy. Swinnen et al [25] present an informative review of existing evidence that the expression and levels of some important lipogenic/cholesterogenic enzymes are upregulated in malignant prostate cells. It is of particular interest that FAS levels are increased in PIN and more so in the adenocarcinomatous glands, which is consistent with the metabolic transformation occurrence early in malignancy. It is apparent that the development and progression of prostate malignancy involves a metabolic shift to de novo lipid biosynthesis.

The available evidence indicates that, in prostate malignant cells, the mitochondrial citrate pool is utilized for oxidation via the Krebs cycle and for export to cytosol for utilization in de novo lipid biosynthesis. This is in contrast to the prevailing concept of tumor cell metabolism of a "truncated Krebs cycle" as proposed by Parlo and Coleman [26,27]. The concept proposes that an increased citrate shuttle activity causes rapid export of citrate; thereby depleting citrate for oxidation by the Krebs cycle. It is well documented that the Krebs cycle downstream reactions from isocitrate to oxalacetate are operational in tumor cells [26-31]. However, the critical reaction is m-aconitase, about which little is known in tumor cells. Kelleher et al [30] and Dietzen and Davis [31] reported the existence of a complete Krebs cycle including citrate oxidation in hepatoma cells. In contrast, Hernanz and de la Fuente [32] reported that the Krebs cycle is truncated in hepatoma cells and concluded that m-aconitase activity was impaired. However, in the presence of m-aconitase activity and isocitrate oxidation, it is not possible that the rate of citrate export could deplete citrate so that essentially no citrate oxidation would occur. The kinetic relationships of citrate export versus m-aconitase activity dictate that, in the presence of a sufficient intramitochondial pool of citrate that would support high citrate export, m-aconitase activity and citrate oxidation will prevail. Therefore, in prostate malignant cells, and likely in other tumor cells, citrate oxidation and citrate export can co-exist as long as sufficient precursors are available for citrate synthesis.

The above description presumes that citrate is the essential source of cytosolic acetyl coA for de novo lipogenesis/cholesterogenesis in malignant prostate cells. However, the possibility exists that acetate derived from circulation might be an alternative precursor for acetyl coA production. This would require the existence of cytosolic acetyl coenzyme A synthetase (AceCS1), which is up-regulated in some lipogenic mammalian cells [33-35]. It is of interest that Pomare et al [36] observed a highly significant ~50% decrease in arterial plasma acetate levels of cancer subjects compared to normal subjects, which suggests that malignant cells might be utilizing acetate from circulation. Hatzivassiliou et al [37] reported the expression of AceCS1 in some malignant cell lines as well as the utilization of acetate. They also showed that inhibition of ATP citrate lyase resulted in >90% inhibition of A549 xenograft tumor growth, but only about 50% inhibition of PC-3 tumor growth; which could be indicative of PC-3 tumor cell utilization of acetate for AcCoA synthesis. Our recent studies (unpublished information) show that AceCS1 is expressed in PC-3 cells in culture and in PC-3 xenograft tumor cells. Therefore the possible utilization of acetate as a supplemental or alternative source of acetyl coA for lipogenesis/cholesterogenesis in prostate nalignancy must be investigated.

### Citrate in the detection of prostate cancer- Magnetic Resonance Spectroscopy Imaging (MRSI)

The following presents a significant translational clinical application of the citrate metabolic relationship. A major current problem involved in prostate cancer (PCa) is the absence of sensitive, accurate, and preferably non-invasive procedures for the diagnosis of PCa. Moreover procedures are needed that will permit the early detection, staging, location, and estimation of the volume of the malignancy; and preferably a mapping of the prostate for follow-up of progression and regression of the malignancy. The unique citrate metabolism relationships of the prostate, coupled with recent developments and technological advancements in 1-H MRSI for the in situ determination of citrate levels, provides an excellent diagnostic/detection procedure which can achieve all these goals. In addition, the mapping of malignant loci permits accurate and precise MRSI guided radiotherapy and chemical seeding in targeting and concentrating the treatment at the malignant tissue. There now exist strong, compelling basic and clinical studies in support of the employment of MRS measurements of citrate and other associated metabolites in the diagnosis of PCa and in the in situ visualization of malignant loci [[38-40]; for reviews].

**Figure 5:**
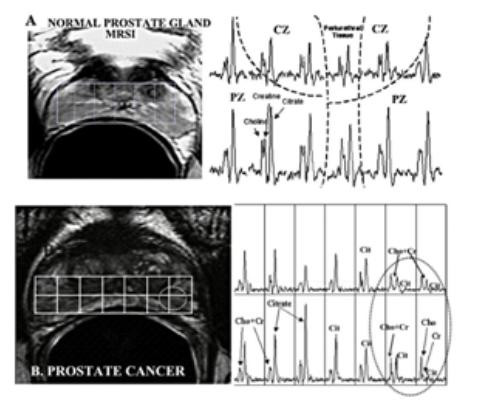
In situ magnetic resonance spectroscopy imaging of prostate gland. Adapted from Kurhanewicz et al [42].

The combination of magnetic resonance imaging (MRI) and MRSI results in a metabolic-anatomic visualization (ie a metabolic map) of the prostate gland. Figure 5 shows the in situ metabolic profile of a normal prostate gland; and also reveals, as we proposed in 1991 [41], that the peripheral zone is the specific region of the prostate that is responsible for the function of citrate production and secretion. In contrast to the uniformly high citrate in normal peripheral zone tissue, the presence of malignant loci exhibits a depletion of citrate. Conversely, the choline and creatine levels are low in normal peripheral zone, and increase in malignant loci. It is likely that the choline increase is due to the requirement for increased membraneogenesis that is essential for growth/proliferation of the malignant cells. This inverse relationship is commonly employed as a ratio for identifying malignant sites in the peripheral zone. MRSI also reveals that glandular BPH involves the proliferation of citrate-producing secretory epithelial cells, presumably from transition zone, into the central zone (figure 5).

**Figure 6:**
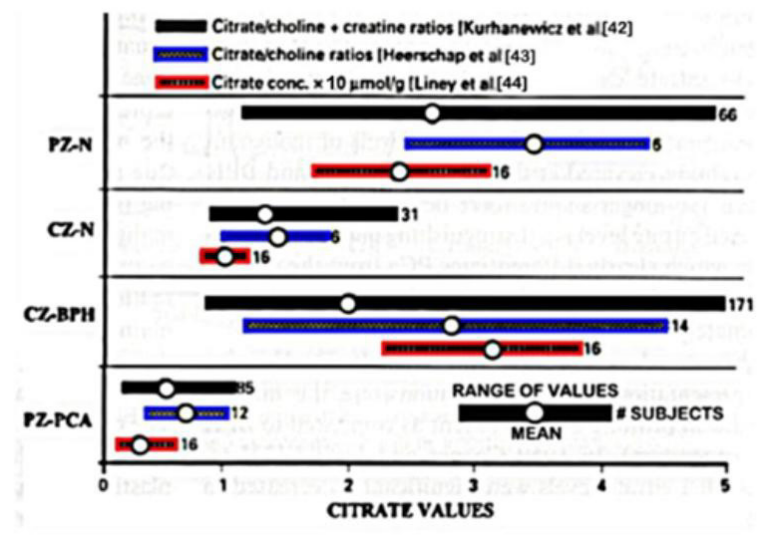
Compilation of in situ MRS citrate analysis of prostate glands from three studies. PZ = peripheral zone; CZ = central zone.

Figure 6 is a composite of early studies from three different laboratories that employed 1-H MRS examination of patient populations [42-44]. The results reflect the striking consistency of the significant decrease in peripheral zone citrate levels of PCa versus normal or BPH prostate. It is notable that none of the PCa subjects from the three separate studies exhibited peripheral zone malignancy that exhibited a high citrate level representative of the normal peripheral zone citrate level. This provides evidence that malignant prostate cells in situ do not exist as citrate-producing cells. This supports the concept that the metabolic transformation is essential for the manifestation of malignant activities of the neoplastic cells.

### Zinc: a tumor suppressor agent in prostate cancer?

**Figure 7:**
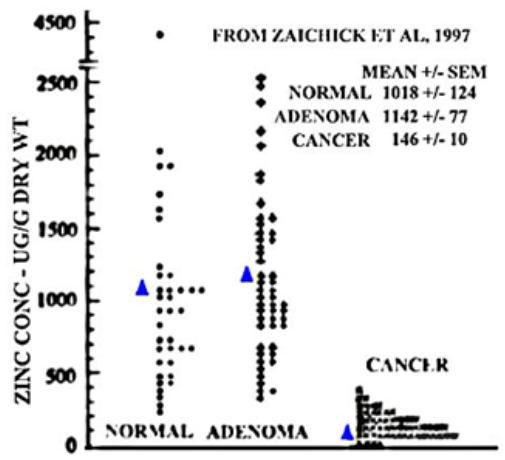
Comparison of zinc levels in normal, benign, and cancer tissue. Each point represents a sample from a different subject.

Any suggestion of tumor suppression in prostate cancer by an agent and/or a gene must have a strong and rational clinical as well as experimental basis. The following enumerates the supporting information regarding zinc suppression of prostate malignancy. As already emphasized, malignant prostate tissue is characterized by a dramatic decrease in zinc level as compared to extremely high zinc levels in normal and hyperplastic glands. Most importantly, malignant tissue rarely, if ever, exhibits the high zinc content that characterizes nonmalignant prostate glands. The consistency of these relationships is further represented in Table 2 that summarizes the results of seventeen separate reports in which zinc levels were compared for normal prostate, BPH, and cancer tissue samples. The studies represent a diversity of tissue collection procedures, a diversity of subjects and clinical conditions, a diversity of zinc analytical methods and calculations, and other such variables. Despites such heterogeneous conditions, a consistent 68% decrease with only a 3% standard error is evident. In contrast, while BPH shows a trend toward an 65% increase in zinc versus normal prostate, the standard error is about 100%; ie a high variability. Figure 7 derived from Zaichick et al [45] shows individual zinc values in prostate tissues obtained from different subjects. The zinc levels in cancer tissue are decreased by ~85% compared to normal tissue values. Most importantly, there is no cancer sample that contains the high zinc levels that characterize normal or BPH levels (i.e. no overlap of the mean +/- sem). It is also significant that the distribution of zinc levels in figure 7 mirrors the observations of citrate changes shown in figure 6. Due to the fact that the decrease in citrate is due to and preceded by the depletion of zinc, the in situ MRS studies are also indirect measurements of changes in zinc in malignancy. All of this evidence is culminated in the in situ study that shows the depletion of zinc in adenocarcinomatous glands as contrasted with accumulated zinc in the glandular epithelium of normal peripheral zone glands [46]. This study also dispels the notion that the decrease in zinc in prostate cancer tissue is due to a decrease in luminal prostatic fluid rather than a decrease in cellular zinc. We submit that all of these observations in combination firmly establish that normal prostate glandular epithelium accumulates high zinc levels; and, conversely, the malignant cells lose the ability to accumulate zinc early in the malignant process.

**Table 2:** ZINC LEVELS IN PROSTATE COMPILED FROM 17 PUBLISHED REPORTS

	**MEAN VALUES**	**% CHANGE FROM**
**NORMAL**		
	**MEAN +/- SEM**	**MEAN (SEM)**
NORMAL	755 +/- 158	---------------------
BPH	1270 +/- 273	+65 (45)% NS
PCA	276 +/- 48	-68 (3)% P<0.001

**% CHANGE FROM NORMAL IS OBTAINED FROM THE SUM OF THE CHANGES OBSERVED IN EACH STUDY THAT INCLUDED NORMAL VALUES (15 REPORTS)**

The inability of malignant cells to accumulate zinc is not, in itself, evidence of a tumor-suppressive effect of zinc. There must be associated effects and consequences of zinc that are inhibitory to the malignant cells. Such anti-tumor consequences do exist and can be characterized as follows.

a. Metabolic/bioenergetic effects. As already described, zinc accumulation in prostate cells truncates the Krebs cycle and inhibits citrate oxidation. In addition, high zinc levels also inhibit the respiration and terminal oxidation of prostate mitochondria [47]. These metabolic effects of zinc have bioenergetic and synthetic consequences as already discussed.

b. Growth/proliferation effects. The accumulation of zinc inhibits growth and proliferation of prostate cells [48]. We reported [49,50] that zinc induces apoptogenesis in prostate cells that results from its direct effect on mitochondrial release of cytochrome c followed by activation of the caspase cascade and ultimately apoptosis. This effect is corroborated by the recent report of Huang et al [51]. Uzzo et al [52] reported that zinc sensitized malignant prostate cells to apoptosis through its inhibition of NF-kB.

c. Invasive/migration effects. Zinc has also been shown to inhibit the invasive capabilities of malignant prostate cells. Ishii et al. [53] reported that the ability of LNCaP cells to invade Matrigel was strongly suppressed by Zn++. In another study Ishii et al. [54] found that aminopeptidase N purified from human prostate was irreversibly inhibited by low concentrations of zinc; which, they concluded, could be associated with invasive capability. Uzzo et al [55] reported that the suppressive effect of zinc on the angiogenic and metastatic potentials of cancer cells was also mediated through the inhibition of specific pathways that regulate progression of prostate cancer.

These effects of zinc accumulation are inhibitory to and incompatible with the prostate malignant process and can be defined as "tumor suppressor" effects of zinc. The depletion of zinc that occurs in malignant prostate cells in situ eliminates these anti-tumor effects. Under these conditions the neoplastic cell is able to meet the metabolic/bioenergetic requirements of malignancy, is able to grow and proliferate, and is able to invade the host tissue and metastasize.

### Zip1: a tumor suppressor gene in prostate cancer?

The critical issue becomes the mechanism involved in the unique ability of normal prostate cells to accumulate high zinc levels; and the mechanism involved in the lost ability of malignant cells to accumulate zinc. For an extensive review of zinc and zinc transporters in prostate cells see [9]. The intracellular level of all cells is firstly dependent upon the existence of zinc uptake transporters to extract zinc from external (e.g. interstitial fluid) sources. Once within the cell, the cellular zinc might be subjected to zinc export from the cell. Until recently, virtually no information existed regarding the mechanisms/processes involved in the accumulation of zinc in prostate cells. Because zinc uptake is the first step in the cellular accumulation of zinc, we had initiated studies to identify this process in prostate cells; and demonstrated that prostate cells contain a rapid zinc-uptake transport process that is operational at low concentrations of zinc representative of plasma zinc levels [56]. We tentatively identified hZIP1 as the zinc uptake transporter associated with this transport process. In pursuant studies, Franklin et al [57] functionally established hZIP1 as the transporter that functions in the uptake and accumulation of zinc in prostate cells. This role of ZIP1 in prostate cells was corroborated by others [51,58].

However, all of the above studies were conducted in prostate culture cells under in vitro conditions. An essential missing link was the relationship in normal and malignant prostate glands in situ. The first clue was derived from the report of Rishii et al [59] who found that ZIP1 expression (determined by RT-in situ-PCR) in prostate glandular epithelium was low in African-American males as compared to Caucasian males. They concluded that this genetic racial difference could account for the higher susceptibility of African-Americans to prostate cancer. In a recent study, the in situ expression and levels of ZIP1 and zinc in malignant versus nonmalignant prostate glands in tissues from prostate cancer subjects were determined [46]. The results demonstrated the consistent down regulation of *ZIP1 *gene expression, the decrease in ZIP1 transporter protein, and the depletion of cellular zinc levels in malignant versus nonmalignant glands. Thus, the clinical and experimental evidence are corroborative of the role of ZIP1 and zinc changes associated with the transformation of normal glandular epithelial cells to malignant cells. Consequently, as long as ZIP1 transport activity exists and results in high zinc accumulation, the tumor suppressor effects of zinc will prevent the malignant activities of the neoplastic cells. **Therefore, we propose that *ZIP1 (SLC39A1) *can be described as a tumor suppressor gene in prostate cancer.**

This leads to the issue of the mechanism of down regulation of *ZIP1 *expression in the neoplastic/malignant cell in situ. In contrast to its down-regulation in the malignant prostate glands in situ, Zip1 is expressed in human prostate malignant and nonmalignant cell lines thus far examined [46,51,56-58]. No information currently exists regarding its expression in metastatic sites in situ. The reappearance of ZIP1 in the cell lines demonstrates the likelihood that its down-regulation in adenocarcinomatous glands is not due to a permanent fatal mutation or gene deletion; but probably results from epigenetic silencing of gene expression under in situ conditions. Consequently, our proposal that *ZIP1 *is a tumor suppressor gene in prostate cancer is similar to the reported involvement of *SLC5A8 *as a tumor suppressor gene in colon and other cancers [60-65]. *SLC5A8 *encodes a monocarboxylic acid transporter protein, and its down regulation results from epigenetic silencing. The specific role of the transporter in tumor suppression is not established. Ganapathy et al [62] propose that the monocarboxylic acid transporter causes the accumulation of butyrate, which has an apoptotic effect that is inhibitory in a variety of tumor cells. As we described, the role of ZIP1 transporter and zinc accumulation as a prostate tumor suppressor is clearly defined and includes apoptotic as well as other tumor suppressor effects. We should not preclude the possibility that, in addition to ZIP1, other zinc transporters could be involved in the loss of zinc in malignant prostate cells. Presently, no strong evidence exists that implicates other zinc transporters in malignant prostate cells in situ in prostate cancer.

### Zinc in the treatment/prevention of prostate cancer: not an enigma

The above discussion leads to a rational expectation that the restoration of zinc accumulation in the malignant prostate cells should arrest the malignancy and cause the death of the tumor cells. Moreover, if zinc accumulation is restored in the neoplastic/premalignant stage (figure 3), overt malignancy should be prevented. How can these expectations be achieved? One must begin with the recognition of two requirements for the efficacious employment of zinc against prostate cancer: 1) the bioavailability of sufficient zinc (i.e. the interstitial fluid zinc) for uptake by the prostate cells; and 2) the availability of a cellular zinc uptake process (e.g. ZIP1). As long as these two requirements are not compromised by the imposition of interfering factors or conditions, the clinical and experimental evidence of the tumor-suppressive effects of zinc should be manifested.

At this point, we cannot ignore the one dot that has not yet been connected; which is the composite results of epidemiologic studies. Several studies that attempt to relate the use of dietary zinc supplementation to prostate cancer have been reported. The results and conclusions of these studies have varied from zinc supplement use having no effect; zinc supplement use being preventative against prostate cancer; and zinc supplement having harmful exacerbating effects on prostate cancer. These studies and the complex and multivariate factors that impact them are the subject of our recent review [11]; and will not be repeated here-in. There is a major concern that must be highlighted. The epidemiologic studies cannot and should not be interpreted as evidence that contradicts the clinically and experimentally established relationships that we have presented. [for review see [11]]. A clear distinction must be made between any effects of dietary zinc supplement use and the direct effects of zinc accumulation on prostate malignancy. The former is complicated by the interaction of known and unknown variables that either compromise the bioavailability of zinc and/or the ability of cells to accumulate zinc. In fact, in the case of reported adverse effects of use of high zinc supplements, the possibility exists that factors other than the bioavailability of zinc are responsible for such effects. There is a consistency in all the reports that the use of moderate levels of zinc supplement does not impose any additional risk; and in some reports could be efficacious against prostate cancer. This is important in the elderly male population in which the bioavailabilty of zinc and its uptake in cells tend to decline with aging [reviewed in [11]]. Therefore, we believe that the prudent use of dietary zinc supplement should be emphasized for this population for general and prostate health reasons. Most importantly, the present inconsistencies of the results from epidemiologic studies do not alter the clinical and experimental consistency of the zinc relationship when the conditions that we described are met.

### Zinc in the treatment/prevention of prostate cancer: potential directions

This provides a basis for consideration of regimens that can achieve the uptake and accumulation of zinc in neoplastic/malignant cells and can provide tumor-suppressive effects against prostate cancer.

a. Genetic manipulation to restore the expression of ZIP1. Presently this is not a practical approach.

b. Genetic manipulation to "activate" expression of alternative zinc uptake transporters. Presently this is not a practical approach.

c. Eliminate or modify the environment conditions of the prostate gland that initiate the epigenetic silencing of Zip1 gene expression. This is dependent upon the elucidation of the mechanism responsible for the down regulation of ZIP1. Once this issue is resolved, this could be a plausible approach.

d. Increase the bioavailability of zinc. This has been the basis for the common use of dietary supplemental zinc as described above. In the elderly male population, supplemental zinc would be efficacious in maintaining a normal plasma zinc level. This should be incorporated into or along with any other interventions. It is also possible that an elevated plasma zinc level could result in zinc uptake by the malignant cells in the absence of ZIP1. This would be dependent upon an elevation of plasma low molecular weight zinc-ligands that become available for cellular uptake [9,11].

e. Incorporation of agents that will deliver and transport zinc into the malignant cells. This is based on the use of ionophores such as sodium pyrithione to deliver cations into cells. The lack of cation specificity and cell specificity might impose toxic effects, which would need to be addressed.

f. Use of m-aconitase inhibitor. A major effect of zinc is its inhibition of m-aconitase activity and citrate oxidation. In its absence, an alternative m-aconitase inhibitor could be employed. Fluoroacetate (→ fluorocitrate) is such an inhibitor. However it is not cell specific and is toxic to other mammalian cells. The development of a prostate cell-specific agent or a cell-specific chaperone for delivery could provide the inhibitory effect on citrate oxidation in the malignant cells.

These are some approaches that are based on the zinc relationships. Additional approaches exist that are based on other aspects of prostate metabolism. For example, inhibition of lipogenic reactions such as ATP citrate lyase [34] and AceCS1 provides potential direction. However, in the case of prostate cancer, more information regarding the genetic/molecular/metabolic relationships of malignancy is essential to such future clinical application.

## Conclusion

The clinical and experimental evidence leads to the following genetic/metabolic relationships in prostate cancer.

1. The unique function of high citrate production and secretion by human prostate glands dictates the metabolic activity and associated genetic requirements of the highly specialized glandular epithelial cells. The unique accumulation of zinc by these cells is a requirement for the achievement of this function.

2. The bioenergetic/synthetic, growth/proliferation, and invasive/migration requirement of malignancy dictates the metabolic activity and associated genetic requirements of the malignant cells.

3. The malignant process requires the genetic and metabolic transformation of normal zinc-accumulating citrate-producing normal cells to citrate-oxidizing malignant cells that lose the ability to accumulate zinc.

4. The accumulation of zinc exhibits tumor-suppressor effects that are incompatible with the process and activities of prostate malignancy.

5. The expression of ZIP1 zinc uptake transporter is responsible for zinc accumulation in prostate cells. ZIP1 is down regulated in the malignant cells in situ, which causes the depletion of zinc and elimination of its tumor-suppressor effects. Therefore we propose that *ZIP1*is a tumor-suppressor gene in prostate cancer.

6. The restoration of high zinc levels in premalignant/malignant prostate cells will arrest and/or abort prostate malignancy.

7. These relationships can serve as the basis for new approaches to the treatment and prevention of prostate cancer.

8. Genetic/metabolic relationships in prostate malignancy are beginning to provide an understanding of the onset and progression of prostate cancer. Much more research in this critical area is required.

## Authors' contributions

RBF and LCC conceived the review, contributed to the writing of the manuscript; and read and approved the final manuscript
